# The Sessional Addresses

**Published:** 1908-10-10

**Authors:** 


### The Sessional Addresses.

Notwithstanding certain indications, notably the complete omission of the opening sessional address at four London medical schools, that this form of oratory is losing popularity with the medical student, there is equal probability that the public at large takes a considerable, and perhaps an increasing, interest in these October speeches. This year there were several addresses of unusual excellence, and the palm is undoubtedly to be awarded to that delivered at the Middlesex Hospital by Mr. Rudyard Kipling, who gave expression to several very plain truths which medical men do not often care to put before the laity themselves, but are none the less matters of some moment. We all know Mr. Kipling's versatility and the extraordinarily wide range of his information on subjects of which the average man is profoundly ignorant; but, though his treatment of the profession in his published work has certainly been indulgent, his doctors are seldom the principal or even the prominent puppets on his stages. There is, it is true, a very vivid description of a case of locomotor ataxia in one of the most intensely tragic of his short stories, but even there the onset and course of the symptoms were, to say the least of it, distinctly anomalous. It might have been supposed, then, that the medical profession bulked less largely in Mr. Kipling's mental vision than his soldiers, his engineers, his Indians, and the other types with whose characterisation he has been so successful. If that is the case, however, at least the image is distinct and accurately focussed; for a more sympathetic and forcible description of the work and aims of a much misunderstood profession than is contained in his address it would not be easy to find. Especially noteworthy, and we are very glad to observe the attention which this passage has attracted in the daily press, are his pronouncements on vivisection, which summed up in one or two burning sentences the general attitude of the ordinary educated man of the world towards this question. It is no mean thing for a body of men engaged in researches prosecuted for the common good of humanity to be able to feel that the general opinion of the intellectual aristocracy of the country is overwhelmingly in their favour, and opposed to their traducers. The gratitude of the profession, and particularly of those members of it who are engaged in experimental research, is due to Mr. Kipling for taking up the cudgels on our behalf, and for wielding them with such vigour and dexterity.

Probably the address which is, after Mr. Rudyard Kipling's, most novel and instructive, is that delivered at the Polyclinic by Dr. Robert Jones on the relations of insanity, wit, and humour. Primitive man is alleged to have been a person devoid of humour, a gift which has been among the latest products of civilisation, and one which is soon removed when the mental functions break down. There is, no doubt, a great deal of truth in the statement that the delusions which appear so laughable to the normal mind are a matter of deadly earnest to the person whom they afflict, and that the absence of altruistic emotions prevents the insane from appreciating humour. But we are not by any means as certain that humour and the power of appreciating it are so entirely a recent development in the evolution of the human race as Dr. Jones wishes us to believe. Of course, the conceptions of children and savages as to what is or is not funny are often very crude compared with those of an educated man, but we doubt very much whether the inhabitants of any civilised country spend as much of their time in laughter as do the inhabitants of Central Africa, for instance. Moreover, there are those who maintain that certain animals manifest a sense of humour of no mean order, and if that contention can be sustained—on which point we offer no opinion—the period at which man or his ancestors developed the gift must be antedated to a much more remote period than that which the superintendent of Claybury Asylum would assign.

